# Prevalence and related factors of intimate partner violence among married women in Garmsar, Iran

**DOI:** 10.5249/jivr.v14i3.1693

**Published:** 2022-07

**Authors:** Tahereh Kamalikhah, Ali Mehri, Farid Gharibi, Nooshin Rouhani-Tonekaboni, Masoume Japelaghi, Elham Dadgar

**Affiliations:** ^*a*^ Student Research Committee, School of Nutrition and Food Sciences, Semnan University of Medical Science, Semnan, Iran.; ^*b*^ Health Education and Promotion Department, Sabzevar University of Medical Science, Sabzevar, Iran.; ^*c*^ Social Determinants of Health Research Center, School of Medicine, Semnan University of Medical Sciences, Semnan, Iran.; ^*d*^ Health Education and Promotion Department, Research Center of Health and Environment, School of Health, Gilan University of Medical Science, Gilan, Iran.; ^*e*^ Department of Nursing, Aligoudarz School of Nursing, Lorestan University of Medical Sciences, Aligoudarz, Iran.

**Keywords:** Mental Health, Domestic Violence, Intimate Partner Violence

## Abstract

**Background:**

Intimate partner violence (IPV) is the most common type of domestic violence often used by men against their wives. Due to the destructive and widespread social and health consequences of IPV, the present study aimed to investigate the prevalence and related factors of IPV among married women in Garmsar, Iran.

**Methods:**

Using multi-stage clusters sampling method, this cross-sectional study included 400 married women in Garmsar, Iran. The data collection process was conducted during October and De-cember 2019 using a researcher-made questionnaire. The content validity of the questionnaire was confirmed using content validity ratio (CVR) and content validity index (CVI) indicators (0.85 and 0.88, respectively). Also, the reliability was confirmed by examining the internal consistency and obtaining a score of 0.93 for Cronbach's alpha. Descriptive and analytical statistics were performed using t-test, analysis of variance (ANOVA), and Tukey’s post-hoc test.

**Results:**

Most participants were in the age range of 20-40 years (mean age: 34.9 years). The overall exposure of women to IPV was 56.11%. In addition, the most prevalent types of IPV included legal (24%), social (24%), financial (22%), verbal (16%), physical (13%), emotional (12%), and sexual (11%). The effective factors on the prevalence of IPV included number of children, education level, occupation, and age (P less than 0.05).

**Conclusions:**

We witnessed that women living in Garmsar faced different types of IPV and their overall exposure to this phenomenon was higher than the national and global average. To resolve the problem, the following measurements are recommended: a careful investigation of the reasons for the spread of IPV, implementing interventions based on reliable evidence, and serious cooperation of the experts and relevant governmental and non-governmental institutions, particularly citizens.

## Introduction

The term violence is defined in the legal literature as "inappropriate, illegal, and aggressive use of power." Violence against women is a phenomenon in which women's rights are violated as they are harassed and abused by men solely due to their gender.^1^ The most common type of violence against women is intimate partner violence (IPV), which is a type of domestic violence.^2^ IPV is an unconventional and annoying behavior intentionally or unintentionally committed by one partner against the other one.^3^ The international statistics show that IPV against women is higher than that of men, with 1.3 million people in the world dying each year as a result of domestic violence (equivalent to 2.5% of global deaths), while 80% of them are female.^4^ The prevalence of female exposure to the IPV is significantly different around the world, varying from 15 to 75%.^3^

In 1988, the Institute of Medicine (IOM) released a IPV causes a higher rate of death and severe health problems in women. Also, if IPV happens at younger ages, it will have more devastating effects.^5^ IPV is the main preventable cause of illness, disability, and death in women.^6^ Exposure of women to IPV leads to physical problems, such as tissue damage, especially to internal organs, fractures, disabilities, inflammatory diseases, infections, complicated pregnancies, chronic head and abdominal and pelvic pain, and sexual dysfunction.^7^ IPV can also cause psychological problems such as loneliness, depression, anxiety, obsession, eating and sleeping disorders, suicide, and low self-esteem.^14^ In addition, violence against women not only violates their social rights but also deprives them of the ability to achieve success and self-fulfillment.^8^

As one of the current global priorities is to ensure that women achieve the highest standards of health and well-being, treaties such as the Sustainable Development Goals (SDG) emphasize gender equality, as well as the empowerment of women and girls while eliminating all forms of violence against women in the world by 2030.^6^ Thus, in many developed countries, domestic violence and IPV against women are important public health issues, and the mechanisms of identifying, referring, and promotional interventions such as social support services, education, counseling, psychoanalysis, and psychotherapy, as well as available relief and asylum centers have been described.^9^

Undoubtedly, the first step in designing and implementing interventions is to assess the situation using field surveys, but domestic violence in different societies, especially in developing countries, is either not reported or is underestimated.^10^ For example, Bangladesh is a highly patriarchal society and marriage is often imposed on women. In this society, violence against women often is not disclosed out of the family environment and women are forced to continue living together even if they are dissatisfied with their marital life due to severe social and cultural pressures.^3^ According to the reports of the World Health Organization (WHO), 55-95% of women experiencing physical and sexual violence have never referred to formal organizations for help.^11^

Numerous factors such as gender-related norms, poverty, lack of access to education, lack of authority, unequal gender attitudes, cultural acceptance of violence against women, cultural and social norms, fear of the consequences of exposing the issue, and economic dependence of women would make this phenomenon hidden.^11^ Therefore, conducting accurate and transparent field studies to determine the prevalence and extent of female exposure to IPV, especially in developing countries, is of prime importance. Accordingly, the present study aimed to investigate the prevalence and related factors of IPV among married women in Garmsar, Iran. 

## Methods 

***Design and Participants***

This cross-sectional study randomly selected 400 married women in Garmsar, Iran from October to December 2019. The sample size was calculated based on the formula n=z (1-a/2)^2^ * pq/r^2^ with 95% confidence interval (CI), p=q=0.5, and r=0.005. The sample size was estimated as 385 people, but we examined 410 people to ensure the adequacy of samples. All married women with at least one year of experience living together were included in the study. 

***Instrument***

The data collection tool was a researcher-made questionnaire, the dimensions and initial questions of which were determined based on similar tools. The content validity of the questionnaire was assessed using content validity ratio (CVR) and content validity index (CVI). All the items of the questionnaire were assessed by ten experts in the fields of psychology, sociology, and health education and promotion based on the four criteria of "necessity", "relevance", "transparency", and "simplicity" in a four-point Likert scale. The results of the "necessity" criterion were used to calculate the CVR and the results of "relevance", "transparency", and "simplicity" criteria were used to calculate the CVI according to the following formula: ^12^

CVR/CVI=(nE- N/2)/(N/2)

Where nE is the number of experts selecting two positive items (such as “fully necessary” and “relatively necessary”) in the scale, and N indicates the total number of experts. The acceptance score of 65% was determined due to the participation of ten experts.^12^ The CVR and CVI indices were approved with scores 0.85 and 0.88, respectively. The face validity of the questionnaire was confirmed through examining the comprehensibility and language use according to the views of ten experts and 20 women from the target group. The reliability of the questionnaire was also assessed by examining internal consistency using a pilot study with a sample of 50 individuals in which a score of 0.93 for Cronbach's alpha confirmed the reliability. The final questionnaire consisted of two main parts encompassing demographic and contextual information, with 58 questions to assess the prevalence of seven dimensions of IPV, including verbal, physical, sextual, financial, social, emotional, and legal. The questionnaire also used 5-point Likert scale to assess different aspects of IPV. 

***Data collection***

The sampling method was based on multi-stage clusters in which the study samples were randomly selected from the defined clusters (geographical zone of the city). In addition, the proportional allocation method was used, indicating the number of samples taken from different clusters according to the proportion of their population from the population in Garmsar. The households survey was used and the data collection method was interviewing. In this process, the interviewers entered the participants' answers to the questionnaire after asking the questions from them in a comprehensible way.

***Statistical analysis***

Both descriptive and analytical anlayses were performed on the data. In the descriptive section, the scores for the options were determined from 0 to 4 based on a 5-point Likert scale (from never to always). The results of the quantitative variables were reported as mean (standard deviation) and the results of the qualitative variables were considered as frequency (percentage). In the analytical part of the study, the relationship between the demographic and contextual variables and the prevalence of different types of IPV was assessed using t-test, analysis of variance (ANOVA), and Tukey’s post-hoc test. The statistical analysis was conducted in SPSS version 19, and a P <0.05 was considered as significant. 

***Ethical considerations***

The ethics committee of Semnan University of Medical Sciences, Iran approved the study protocol (code: IR.SEMUMS.REC.1396.259). Participation in the study was done in a completely voluntary manner, an informed consent was obtained from all participants prior to the study, and we kept the participants’ identity confidential.

## Results

In this study, the response rate of participants to the questionnaire was 86.4%. Most participants were in the age range of 20-40 years (average age: 34.9 years), and the age range of their partners was 30-50 years (average age difference: 5.3 years). The majority of participants had one to two children and the average marriage period was 14.82 years. Women had a higher education level compared to their partners (45.6% vs. 39%). Howevr, the employment rate of women was significantly lower than that of men (16.8% vs. 25.5%). Most marriages were non-familial, and in most cases (55%) families played a major role in choosing the partner. 

The results showed that the overall exposure of women to IPV was 56%. Also, while legal IPV had the highest prevalence (24%), sexual harassment had the lowest prevalence (11%) (see Table 1 ). 

**Table 1:** The prevalence of different types of IPV in Garmsar (in percentage).

Dimentions	Minimum	Maximum	Mean	SD deviation	95% Confidence Interval	
					Lower	Upper
Verbal	9	44	16	7	14.87	16.23
Physical	11	53	13	5	12.15	13.15
Sextual	9	45	11	4	10.63	11.40
Financial	17	83	22	11	21.21	23.31
Social	17	75	24	11	22.71	24.90
Emotional	8	36	12	6	11.27	12.51
Legal	0	74	24	48	19.52	28.89
Total	12	77	56	18	51.20	63.74

We witnessed that factors, including the number of children, woman’s education level, partner’s education level, partner’s occupation status, and age difference had a significant effect on some dimensions of IPV. According to our results, the variable of the number of children was correlated with the prevalence of verbal, financial, social, emotional, and legal IPV; the education level of spouses was linked with the prevalence of verbal, physical, sexual, emotional, and legal IPV; the variable of partner’s occupation status was connected with the prevalence of verbal and emotional IPV; and the variable of age difference had a statistically significant relationship with the prevalence of physical IPV (P <0.05) (see Table 2 ).

**Table 2:** The statistical relationship between demographic/contextual variables and the prevalence of different types of IPV (P-value).

	Verbal	Physical	Sexual	Financial	Social	Emotional	Legal
Woman’s age	0.091	0.138	0.965	0.774	0.368	0.595	0.623
Partner’s age	0.279	0.701	0.734	0.944	0.651	0.881	0.928
Number of children	0.004*	0.189	0.178	0.015*	0.012*	0.006*	0.020*
Years of living together	0.107	0.408	0.952	0.761	0.888	0.828	0.733
Woman’s education status	0.019*	0.000*	0.028*	0.654	0.570	0.030*	0.031*
Partner’s education status	0.003*	0.002*	0.012*	0.102	0.492	0.034*	0.001*
Woman’s job status	0.716	0.268	0.267	0.970	0.662	0.983	0.833
Partner’s job status	0.014*	0.150	0.231	0.078	0.059	0.016*	0.132
Type of marriage	0.992	0.579	0.983	0.412	0.220	0.787	0.530
Playing a key role in choosing husband	0.514	0.870	0.813	0.712	0.541	0.973	0.625
Age difference	0.130	0.040*	0.106	0.210	0.077	0.233	0.081
Wife older than husband	0.555	0.304	0.742	0.548	0.598	0.569	0.684

* Statistical significance at the level of 0.05.

The Tuckey’s post-hoc test was used to examine the statistical relationship between the defined categories in demographic and contextual variables with the dimensions of IPV (see Table 3 ). As can be seen, the prevalence of verbal IPV in families with no child was significantly less than those with one to two children. The prevalence of social, emotional, and legal IPV in people with one to two children was significantly higher than people with three to four children. Moreover, physical IPV in couples with an age difference of 6-10 years was significantly lower than couples with an age difference of more than ten years. 

**Table 3:** The relationship between the categories of demographic and contextual variables and the IPV types.

Contextual variables	IPV dimension	Base group (I)	Comparison group (J)	Significant level	Std. Error	Mean difference (I-J)
Number of children	Verbal	Without child	1-2 children	0.015	0.012	-0.037
	Social	1-2 children	3-4 children	0.012	0.014	0.043
	Emotional	1-2 children	No children	0.049	0.011	0.028
			3-4 children	0.025	0.008	0.023
	Legal	1-2 children	3-4 children	0.031	0.061	0.169
Age difference	Physical	6-10 years	More than 10 years	0.031	0.011	-0.027
Woman's education level	Verbal	Elementary	High school and diploma	0.030	0.013	0.035
	Physical	Elementary	High school and diploma	0.001	0.009	0.033
			University degree	0.003	0.009	0.030
	Physical	Secondary school	High school and diploma	0.042	0.009	0.024
	Sexual	Secondary school	High school and diploma	0.021	0.007	0.020
	Emotional	Secondary school	High school and diploma	0.042	0.011	0.030
Partner's education level	Verbal	Elementary	Secondary school	0.014	0.015	0.045
			High school and diploma	0.029	0.012	0.034
			Academic	0.002	0.012	0.045
	Physical	Elementary	Secondary school	0.005	0.011	0.035
			High school and diploma	0.004	0.009	0.030
			Academic	0.003	0.009	0.030
	Sexual	Elementary	Secondary school	0.049	0.009	0.021
			High school and diploma	0.017	0.007	0.020
			University degree	0.007	0.007	0.022
	Emotional	Elementary	University degree	0.018	0.010	0.033
	Legal	Elementary	Secondary school	0.005	0.102	0.0345
			High school and diploma	0.005	0.084	0.281
			University degree	0.001	0.084	0.330
Partner's occupation status	Verbal	Employee	Jobless, worker, etc.	0.011	0.012	-0.034
	Emotional	Employee	Self- employment	0.013	0.007	-0.021

Moreover, women with a primary education level experienced a significantly higher IPV than women with secondary education and diploma, whereas women with primary education experienced a higher level of physical harassment compared to women with high school education, diploma, and university education. In addition, women with secondary school education were more likely to be physically, sexually, and emotionally abused than women with high school and college education. 

The prevalence of verbal, physical, sexual, emotional, and legal IPV in women whose husbands had primary education was significantly higher than women whose husbands had secondary, high school, diploma, or university education. 

Finally, the incidence of verbal and emotional harassment was significantly higher among women with employed husbands compared to women whose husbands were laborers, unemployed, or self-employed.

## Discussion

According to the results of this study, more than half of the participants (56%) had experienced some type(s) of IPV in the past one year. The rate of women’s exposure to IPV is quite different in various parts of the world due to the wide range of cultural, social, and economic factors. While the prevalence of women experiencing IPV in the world is about 30%,^8^ this rate is about 26% in the Middle East,^13^ and 36% in most African countries.^8^ There are significant differences among African countries, with 42.7% in Zimbabwe,^8^ 64.6% in Ethiopia,^14^ 33.6% in Ghana, ^4^ and 35.5% in East Sudan.^15^ The prevalence of women experiencing IPV in Asian countries also significantly varies, with 30.4% in Turkey^16^ and 63.83% in Pakistan;^17^ however, the prevalence of IPV in Sweden as a European country is about 20%.^18^

Studies conducted in Iran on women's exposure to IPV showed a prevalence of 54.5% in Shiraz,^2^ 38.7% in Tehran and Hashtgerd,^19^ and 50.9% in Rafsanjan.^20^ The results of a systematic review and meta-analysis estimated the rate of domestic violence against Iranian women at 45.33%.^21^ however, the results of the present study showed that the prevalence of IPV in Garmsar was higher (56%) than the national and global averages (45.33% and 30%, respectively).

The findings of this study also showed that the most prevalent types of IPV included legal (24%), social (24%), financial (22%), verbal (16%), physical (13%), emotional (12%), and sexual (11%). Nevertheless, physical abuse in Ethiopia,^22^ psychological harassment in Nepal,^22^ verbal harassment in Sudan^15^ and sexual harassment in Thailand^23^ had the highest prevalence. In the neighboring countries of Iran, psychological, verbal, and emotional IPVs were more prevalent than other types. For example, the prevalence of psychological/verbal, physical, and sexual IPVs in Turkey was 54.5%, 30.4%, and 6.3%, respectively.^16^ Furthermore, the prevalence of emotional, physical, and sexual IPVs in Pakistan was 81.8%, 56.3%, and 53.4%, respectively.^17^

In general, the prevalence of emotional IPV is higher than other types of IPV in the world, which can be attributed to men's lack of knowledge about this type of violence due to lack of adequate training and the existing difficulties in proving IPV in possible lawsuits. The study of trends also showed a decrease in the prevalence of sexual and physical IPVs and an increase in emotional IPV.^21^

In other Iranian studies, the rate of psychological-emotional IPV was often more than that of physical IPV, and the rate of physical IPV was more than that of sexual IPV. For example, the exposure of women living in Jahrom to physical, sexual, and emotional/psychological IPVs was 16.4%, 18.6%, and 44.4%, respectively.^24^ The prevalence of psychological, physical, and sexual abuse in Shiraz was approximately 52%, 18.2%, 14%, respectively.^2^ the prevalence of physical and psychological abuse among women living in Safadasht was 58.5% and 83%, respectively.^25^ In another study, the prevalence of physical, verbal, emotional, and sexual abuse in Rafsanjan was 23.1%, 38.1%, 21.3%, and 18.9%, respectively.^20^ Also, based on the results of a systematic review and meta-analysis, the prevalence of emotional, physical, and sexual violence in Iran was 59%, 45%, and 32%, respectively.^21^

In data analysis, only the variables of number of children, woman’s education level, partner's education level, partner's occupation status, and the age difference of couples had a statistically significant relationship with some dimensions of IPV. The lower prevalence of verbal IPV in couples with no child compared to couples with one to two children can be due to the more intense love of couples in their early years of marriage, spending all their time together at home, and the lack of disagreement over raising children in childless couples.

The higher prevalence of verbal IPV in couples with one to two children than couples with three to four children and more can be attributed to the impact of the first child or children on reducing the number and quality of marital privacy, having less companionship with each other, or having disagreements and even disputes with each other over the methods of child care and parenting, which will be gradually improved by gaining more parental experiences and reaching more compatible spousal relationships.

The inverse statistical relationship between the couples' level of education with the prevalence of IPV can be related to the increase in the trainability of couples with higher education and better understanding of their own rights and those of their partners. 

The high level of physical IPV in couples with a higher age difference can be attributed to differences in their perceptions as they belong to two different generations. 

The low prevalence of verbal and emotional IPV in women with working partners can be related to their high level of education and their sufficient and stable income, because many arguments between couples are rooted in their economic problems. 

A review of similar studies showed that while alcohol consumption, criminal record, and the partner's occupation (being a farmer) were effective on the rate of IPV in Ethiopia,^14^ factors such as partner's occupation, alcohol consumption in couples, a record of IPV in women's parents, and polygamy were effective on the high incidence of IPV in Zimbabwe.^8^ In Ghana, a history of beating mother by father led to an increase in the risk of IPV by 41%. In this country women married to men with higher education are 48% less than women married to less literate and illiterate men. In addition, women whose husbands consume alcohol are 2.5 times more likely to be abused by their partners than other women.^4^ In Sudan, factors such as the level of education, polygamy, and alcohol consumption by the husband also contributed to the prevalence of IPV.^15^

In Jahrom, Iran, the variables of couple's age, the elapsed time from marriage, and couple's education level were the factors influencing the occurrence of IPV.^24^ In Shiraz, factors such as the age of the partner, high number of children, tenancy, insufficient monthly income, and the history of IPV in the paternal family of the couple were effective on the prevalence of IPV.^2^ In Rafsanjan, the prevalence of IPV was related to the couple's age, wife's education, husband's occupation, the history of mental illness in the couple, the number of the husband's marriage, and the presence of a disabled child in the family.^20^

IPV is a major global health issue and one of the leading causes of death and disability in women aged 15-44 years.^26^ Given that the main cause of IPV can be traced to gender inequalities in society, it is expected that IPV in civilized societies is less prevalent than developing societies. According to the studies, 77.3% of cases in Jordan are against women and 22.7% are against men,^26^ while IPV among women and men in Sweden is equal with the prevalence rate of 20%.^18^ Violence is created through education, childhood patterns in the family, school, peer group, and the media. Institutionalized gender discrimination in the society due to the dominant power of men in legislation, economy, as well as executive and regulatory institutions have a significant impact on the spread of IPV.^27^ Therefore, the gradual elimination of gender inequalities, life skills training, especially in the marital dimension and conflict management, providing practical counseling, screening women at risk, offering psychotherapy to those in need of treatment, and finally providing legal and social support can be effective in modifying and eliminating IPV.^27^

The main strength of this study is the comprehensiveness of the tools used for addressing all aspects of IPV. However, one of the limitations of this study was that 14% of participants did not answer the questions because of cultural and societal restrictions. It seems that different results would probably have been obtained with the full and free participation of individuals in the study. Another limitation was the incompleteness of the explored dimensions of IPV in the tools of other studies, which impeded the possibility of comparing those findings with the results of the present study. Furthermore, this study was conducted before the COVID-19 pandemic, but several studies showed that the prevalence of IPV has increased after the pandemic.^13^

The findings of the present study showed that women living in Garmsar faced different types of IPV and their overall exposure to this phenomenon was higher than the national and global average. To resolve the problem, the following measurements are recommended: a careful investigation of the reasons for the spread of IPV, implementing interventions based on reliable evidence, and serious cooperation of the experts and relevant governmental and non-governmental institutions, particularly citizens. 

**Acknowledgments**

We would like to express our sincere gratitude to all participants for their assistance. We also thank the Vice Chancellor of Research and Technology at Semnan University of Medical Sciences for providing the financial support.
